# Silk Fibroin-Based Shape-Memory Organohydrogels with Semicrystalline Microinclusions

**DOI:** 10.1021/acsabm.3c00017

**Published:** 2023-03-16

**Authors:** Cigdem Buse Oral, Berkant Yetiskin, Canan Cil, Fatma Nese Kok, Oguz Okay

**Affiliations:** † Department of Chemistry, Istanbul Technical University, Maslak, Istanbul 34469, Turkey; ‡ Department of Molecular Biology and Genetics, Istanbul Technical University, Maslak, Istanbul 34469, Turkey

**Keywords:** organohydrogels, emulsion, self-emulsifier, silk fibroin, *n*-octadecyl acrylate, shape memory

## Abstract

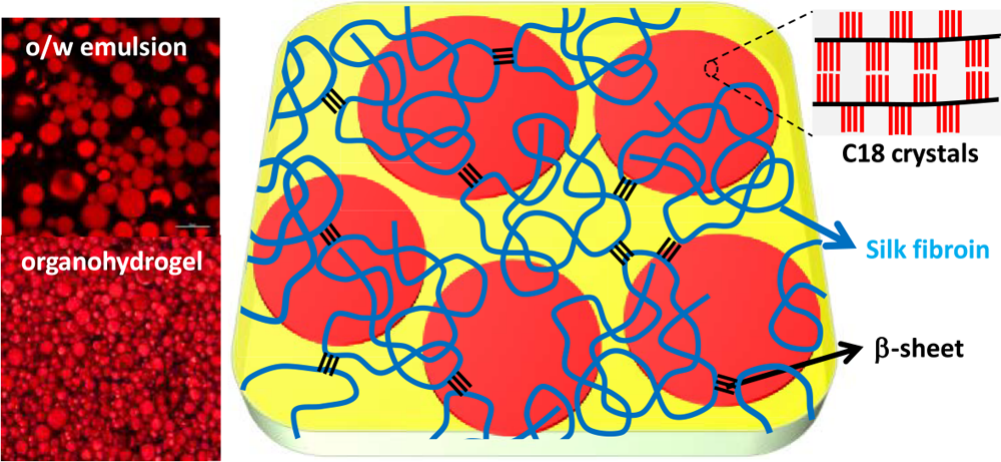

Inspired by nature, we designed organohydrogels (OHGs) consisting of a silk fibroin (SF) hydrogel as the continuous phase and the hydrophobic microinclusions based on semicrystalline poly(*n*-octadecyl acrylate) (PC18A) as the dispersed phase. SF acts as a self-emulsifier to obtain oil-in-water emulsions, and hence, it is a versatile and green alternative to chemical emulsifiers. We first prepared a stable oil-in-water emulsion without an external emulsifier by dispersing the *n*-octadecyl acrylate (C18A) monomer in an aqueous SF solution. To stabilize the emulsions for longer times, gelation in the continuous SF phase was induced by the addition of ethanol, which is known to trigger the conformational transition in SF from random coil to β-sheet structures. In the second step, in situ polymerization of C18A droplets in the emulsion system was conducted under UV light in the presence of a photoinitiator to obtain high-strength OHGs with shape-memory function, and good cytocompatibility. The incorporation of hydrophilic *N*,*N*-dimethylacrylamide and noncrystallizable hydrophobic lauryl methacrylate units in the hydrogel and organogel phases of OHGs, respectively, further improved their mechanical and shape-memory properties. The shape-memory OHGs presented here exhibit switchable viscoelasticity and mechanics, a high Young’s modulus (up to 4.3 ± 0.1 MPa), compressive strength (up to 2.5 ± 0.1 MPa), and toughness (up to 0.68 MPa).

## 1 Introduction

In nature, living organisms develop certain vital strategies for adaptation to surroundings and hence to increase their survival chance.^1−4^ One of the survival strategies of living organisms is the coexistence of hydrophilic and lyophilic components.^5,6^ Cell membrane and antifreezing proteins are examples of structures possessing hydrophilic and lyophilic structures together. All vital cellular processes such as cell–cell recognition occur with the help of an amphiphilic cellular membrane. Antifreeze proteins provide an advantage to living organisms to survive in subzero temperatures.^1^ Inspired by nature, organohydrogels (OHGs) are designed as a new class of polymeric gels combining the advantageous properties of hydrogels and organogels, while their deficiencies are overcome by using them together.^7−9^ The existence of the antagonistic features in the same microenvironment of OHG provides smart functions such as antifreezing,^10^ sensors of temperature,^11^ strain,^12^ and humidity,^13^ shape-memory,^14^ soft robotics,^15^ self-healing,^16^ and signal transmission.^17^

In the past years, several strategies have been developed to obtain OHGs with varying microstructures and dimensions of the antagonistic phases.^7−9^ For instance, Gao et al. prepared freeze-tolerant OHGs with a heteronetwork structure by immersing a chemically cross-linked hydrophilic polymer network in a common solvent containing a hydrophobic monomer followed by in situ polymerization.^18^ Similarly, antifreezing, conductive, self-healing OHGs were prepared by physical cross-linking of aqueous poly(vinyl alcohol) solutions containing an antifreezing binary solvent system.^19^ Recently, cryogelation technology was used by Yetiskin et al. to create OHGs with switching mechanics and viscoelasticity.^20^ They utilized a mechanically robust hydrophilic cryogel scaffold based on silk fibroin (SF) as the continuous phase of OHG, while the macropores of the scaffold acted as the reaction loci for the formation of organogel microinclusions. Another strategy is to prepare emulsion-based OHGs by dispersing hydrophobic monomer droplets and/or alkanes in a continuous aqueous phase containing hydrophilic monomers and an emulsion stabilizer such as surfactants, followed by polymerization.^7,8^ Zhang et al. prepared emulsion-based OHGs with energy storage capacity by dispersing oil droplets in an aqueous solution containing bacterial cellulose nanofibrils as a stabilizer and sodium alginate, followed by adding calcium ions to the emulsion system to form the alginate polymer network as the continuous phase of OHG.^21^ They sustain compressive stresses up to 35 kPa and exhibit an elastic modulus was around 72 kPa. Zhao et al. prepared shape-memory OHGs by in situ polymerization of an emulsion system containing paraffin and hydrophobic monomers as the dispersed oil phase, *N*,*N*-dimethyl acrylamide monomer, and a nanoclay stabilizer as the continuous aqueous phase.^14^

SF is one of the most significant and popular proteins used in biomedical fields because of its outstanding mechanical properties, cell and tissue compatibility, biodegradability, and resistance to various external stimuli.^22,23^ These superior properties make SF favorable for the fabrication of various biomaterials. SF can also be considered as a natural amphiphilic multiblock copolymer due to the existence of both hydrophilic and hydrophobic blocks in its structure. It was shown that SF prevents the coalescence of hydrophobic droplets in aqueous solutions and facilitates the formation of a stable oil-in-water emulsion.^24−28^ Wen et al. used SF as a self-emulsifier to form an oil-in-water (o/w) emulsion by emulsifying 1-butanol in an aqueous SF solution without the need for any other surface-active agent.^25^ Emulsifying performance of SF in combination with a cosurfactant was also used to prepare a double-network hydrogel consisting of a physically cross-linked SF hydrogel and hydrophobically modified polyacrylamide as the first and second networks, respectively.^29^

**Scheme 1:**
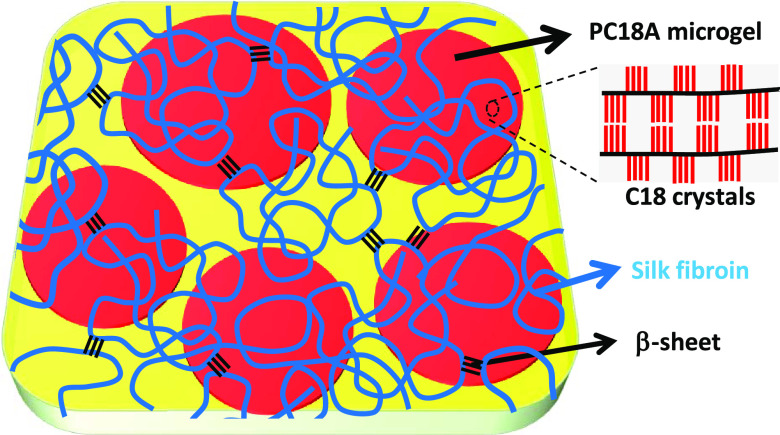
Cartoon Showing the Organohydrogel Consisting of SF Hydrogel Containing Semicrystalline PC18A Microgel Inclusions

Herein, we present a versatile strategy to prepare OHGs consisting of an SF hydrogel as the continuous phase and microinclusions based on poly(*n*-octadecyl acrylate) (PC18A), which is a semicrystalline polymer with a melting temperature of around 50 °C (Scheme 1). Recent works from our group show that the hydrogels and polymer blends based on PC18A exhibit superior properties including switchable mechanics and viscoelasticity, shape-memory, and self-healing functions.^30−32^ The use of aqueous SF solution as the continuous phase of OHG and as a self-emulsifier for the organic microinclusions has not been reported before. The advantage of SF in the preparation of OHG is threefold. First, SF acts as a self-emulsifier to obtain oil-in-water emulsion systems, and hence, it is a versatile and green alternative to chemical emulsifiers. Second, SF provides stability to the emulsion system by inducing β-sheet formation in SF increasing its hydrophobic character and acting as a physical cross-linker. Third, SF is as mentioned above one of the most widely used proteins in biomedical fields.

The OHGs were prepared in two steps. In the first step, a stable oil-in-water emulsion without an external emulsifier was prepared by dispersing the *n*-octadecyl acrylate (C18A) monomer in an aqueous SF solution. To stabilize the emulsions for longer times, gelation in the continuous SF phase was induced by the addition of ethanol which is known to trigger the conformational transition in SF from random coil to β-sheet structures.^33^ In the second step, in situ polymerization of C18A droplets in the emulsion system was conducted under UV light in the presence of a photoinitiator to obtain high-strength OHGs with switchable viscoelasticity, shape-memory function, and a good cytocompatibility. The incorporation of hydrophilic *N*,*N*-dimethylacrylamide and noncrystallizable hydrophobic lauryl methacrylate units in the hydrogel and organogel phases of OHGs, respectively, further improved their mechanical and shape-memory properties.

## 2 Results and Discussion

The formation of a stable oil-in-water emulsion system is of vital importance for the successful preparation of OHGs. In the first subsection, we discuss the formation conditions of an emulsifier-free emulsion of *n*-octadecyl acrylate (C18A) droplets in a continuous aqueous SF solution. The fabrication of OHGs by UV polymerization of the emulsions and their modifications by incorporating hydrophilic and noncrystallizable hydrophobic units in the hydrogel and organogel phases of OHGs, respectively, are discussed in the following subsections.

### 2.1 Emulsification Performance of SF

**Scheme 2:**
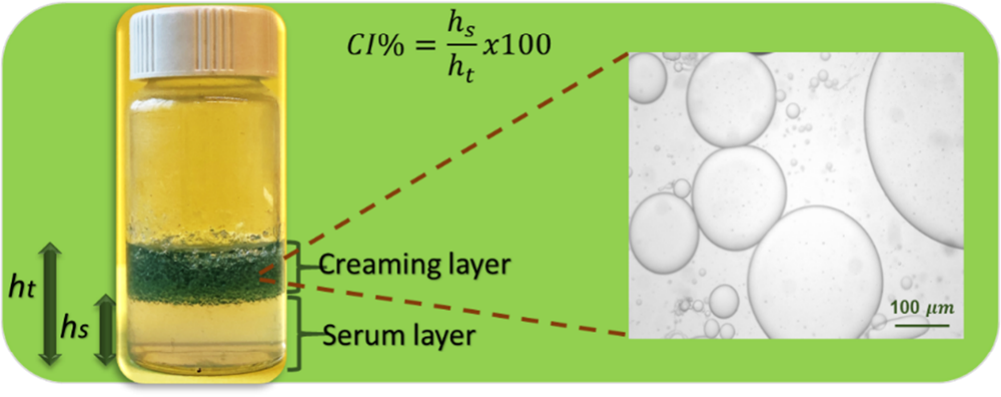
Image of a Mixture of C18A and Aqueous SF Just after Preparation SF = 5%. o/w volume ratio = 4/6. For clarity, C18A was colored with a phthalocyanine. The inset shows the optical microscopy image of the creaming layer

The emulsification performance of SF protein was investigated by using the C18A monomer as the oil phase that was dispersed in an aqueous SF solution to obtain oil-in-water emulsions. To find the optimum conditions for the emulsion system, various experimental parameters affecting the emulsion stability was investigated, including the concentrations of SF (in w/v %) and ethanol (in vol %) in the continuous aqueous phase and the volume ratio of oil-to-water phase (o/w). Hereafter, the concentrations of SF and ethanol were abbreviated simply as %. The stability of the emulsions and the size distribution of the emulsion droplets were monitored by creaming index (CI) and optical microscopy measurements, respectively. CI was calculated using the equation,
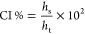
1where *h*_s_ is the height of the clear liquid (serum) layer at the bottom, and *h*_t_ is the total height of the creaming and serum layers (Scheme 2). Thus, CI = 0 means the formation of oil-in-water emulsion without a serum layer while CI = 1 corresponds to a phase-separated solution.

**Figure 1:**
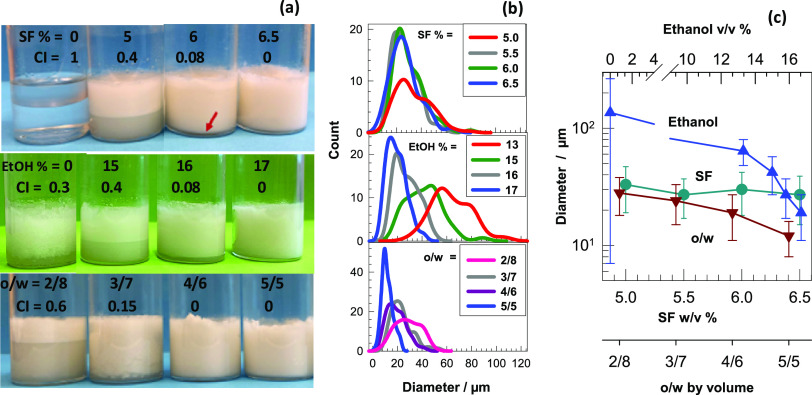
(a, b) Optical images (a) and droplet size distributions (b) of the emulsions formed at various concentrations of SF (upper panel), ethanol (middle panel), and o/w volume ratios (bottom panel). CI values are indicated on the vials in (a). The red arrow in the upper panel in (a) indicates the tiny serum layer at 6% SF The images and size distributions were recorded after 24 h while CI values were measured after 1 week of the emulsion preparation. (c) Average droplet diameters of the emulsions plotted against the concentrations of SF (circles) and ethanol (triangles up), and o/w ratio (triangles down).

In the first set of experiments, SF concentration was varied at fixed ethanol content (17%) and the o/w volume ratio (4/6) (Table S1). The upper panel in Figure 1a shows the images of the emulsions formed at various SF concentrations, as indicated on the vials together with the CI values. In the absence of SF, CI equals units, i.e., C18A forms a separate phase, while in the presence of SF, CI gradually decreases and becomes zero at 6.5% SF, indicating the formation of a stable emulsion. Increasing emulsion stability with an increasing amount of SF is attributed to the increasing interfacial coverage surrounding C18A droplets.^28^ We should note that at SF concentrations higher than 6.5%, gelation in the emulsions occurred immediately after the addition of C18A before the complete dispersion of C18A droplets in the continuous aqueous phase. Thus, the optimum SF concentration is 6.5% because neither early gelation nor phase separation occurred. This emulsion system turned into an SF hydrogel 15 min after its preparation so that the emulsion stability was protected until the start of UV polymerization (Figure S1).

**Figure 2:**
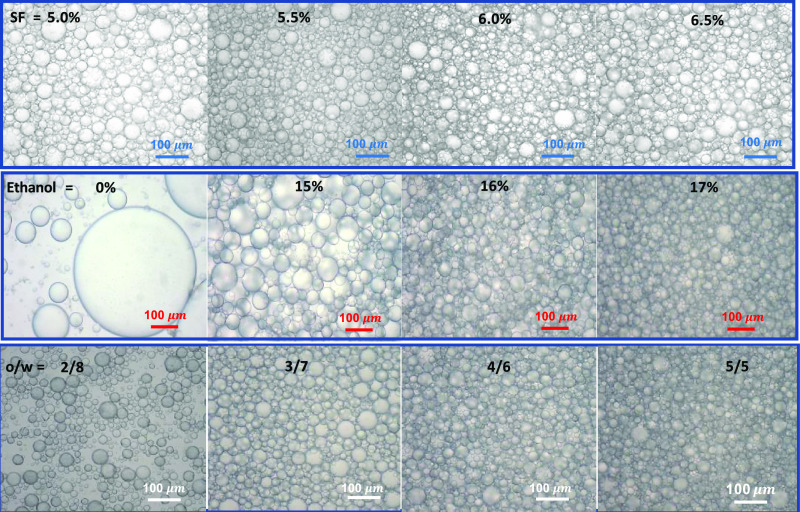
Optical images of the emulsions formed at various concentrations of SF (upper panel), ethanol (middle panel), and o/w ratios (bottom panel). The images were taken after 24 h of the emulsion preparation. The scale bars: 100 μm.

In the second and third sets of experiments, SF concentration was fixed at 6.5%, while the ethanol content of the continuous phase and the o/w ratio were varied (Tables S2 and S3). The images in the middle and bottom panels in Figure 1a show the effects of ethanol and the o/w volume ratio, respectively, on the emulsion stability. At or below 15% ethanol, phase-separated emulsions with a CI value ≤0.4 were obtained, while 17% ethanol creates a stable milky white emulsion without a serum layer. The effect of ethanol on the emulsion stability is due to the increasing β-sheet content making SF more hydrophobic (see Section 2.2).^28^ Moreover, raising the o/w volume ratio also improves the emulsion stability which is attributed to the increased viscosity with an increasing amount of droplets in the emulsion. For instance, a phase separation was observed at o/w = 2/8 and 3/7 with CI values of 0.6 and 0.15, respectively, while a further increase in the oil phase volume fraction leads to a stable emulsion without a separated phase. The size distribution of the droplets was determined by measuring the diameter of the randomly selected 100 droplets using an optical microscope. Figure 2 presents the optical microscopy images of the emulsions of various compositions, while Figure 1b shows the size distribution of C18A droplets in the emulsions. The diameter of the droplets varies between 12 and 136 μm that can be tuned by changing the preparation conditions. Highly polydisperse droplets are obtained at low SF or ethanol concentrations, or at a low o/w volume ratio, as shown by the red curves in Figure 1b. In contrast, quite monodisperse droplets with a polydispersity index of around 1.1 could be obtained at the highest SF and ethanol concentrations and the o/w volume ratio (blue curves in Figure 1b).

**Figure 3:**
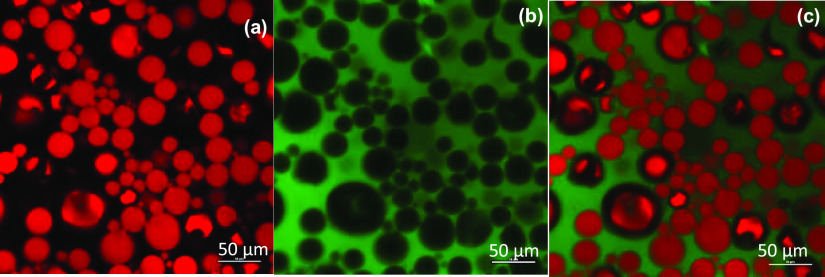
Confocal microscopy images of the emulsion of C18A droplets in an aqueous SF solution. SF = 6.5%. Ethanol = 17%. o/w = 5/5. The continuous aqueous phase was stained using FITC, while the oil (C18A) phase was stained with Nile red. Note that FITC is easily soluble in an aqueous SF solution containing ethanol. The images show the oil (a) and aqueous phases (b), and emulsion (c). Magnification: 50 μm.

Figure 1c shows the average diameter of 100 randomly selected droplets plotted against the synthesis parameters. At a fixed ethanol content (17%) and o/w ratio (4/6), the diameter of the droplets remains at 30 ± 3 μm almost independent of the SF concentration investigated. Moreover, in the absence of ethanol, polydisperse droplets with an average diameter of 136 ± 129 μm form, while the addition of ethanol decreases the droplet diameter significantly, and it becomes 19 ± 8 μm at 17% ethanol. The increasing volume ratio o/w from 2/8 to 5/5 also decreases the diameter from 28 ± 10 to 12 ± 4 μm, and simultaneously, the size distribution becomes narrower. Thus, the optimum parameters to obtain a stable emulsion of C18A droplets with a diameter of 12 ± 4 μm are an aqueous 6.5% SF solution containing 17% ethanol in which C18A is dispersed at an o/w volume ratio of 5/5. Figure 3 shows confocal laser scanning microscopy (CLSM) images of this emulsion system where the continuous aqueous phase was stained using fluorescein isothiocyanate (FITC), while the dispersed C18A phase was stained with Nile red.

### 2.2 OHGs: SF Hydrogel with PC18A Microinclusions

**Figure 4:**
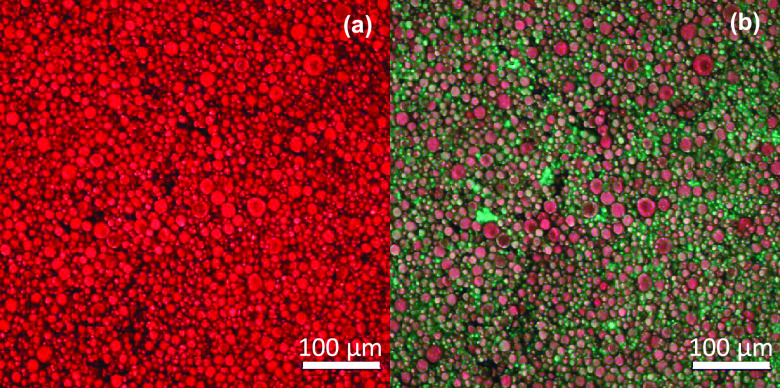
(a, b) Confocal microscopy images of OHG. The images show the dispersed PC18A phase (a) and both phases (b). The PC18A phase was stained with Nile red, while the continuous hydrogel phase was stained using FITC. Scale bars: 100 μm.

The OHG was prepared by adding the oil phase composed of C18A monomer and Irgacure 2959 photoinitiator (0.2 mole% of the monomer) into the optimized SF solution described above to obtain a milky white emulsion, followed by UV polymerization at 23 ± 2 °C for 24 h (Table S4). The OHG thus obtained was extracted with ethanol to remove the unreacted C18A, and then immersed in an excess of water until swelling equilibrium at which it contained 47 ± 1% water. For comparison, a blank sample was also prepared under the same condition except that the UV initiator was not added to the oil phase, and the emulsion system was incubated at 25 °C for 24 h to induce fibroin gelation in the continuous phase. In contrast to the OHG exhibiting a twofold weight swelling in water with respect to its dry state, the blank specimen swells 24-fold which we attribute to the extraction of the dispersed C18A in ethanol leaving voids (pores) behind (Figure S2). Indeed, the gel fraction *W*_g_ for the blank specimen was found to be around 0.1, i.e., 90% of the specimen dissolves in ethanol. Assuming that all C18A monomers are extracted in ethanol, the *W*_g_ value was calculated as 0.08 revealing that only a tiny fraction of C18A remains in the blank sample. In contrast, OHG specimens exhibit a gel fraction *W*_g_ of around unity (1.01 ± 0.03) reflecting that C18A in the droplets was completely polymerized and incorporated as microgels into the 3D SF network of OHG. The CLSM images of an OHG specimen given in Figure 4 reflect that the morphology of the initial emulsion system remains almost unchanged after polymerization.

**Figure 5:**
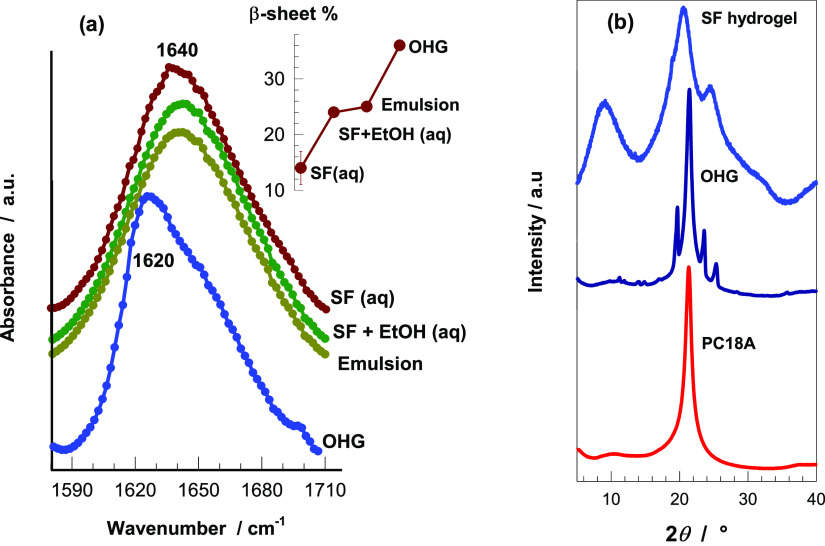
(a) Amide *I* region of FTIR spectra for aqueous solutions of SF, and SF + ethanol, emulsion system, and OHG. The inset shows their β-sheet contents. (b) X-ray diffraction patterns of SF hydrogel, OHG, and PC18A.

The conformational change of SF during the preparation of the emulsion and after UV polymerization was investigated by FTIR and XRD measurements. Figure 5a shows the Amide *I* region of the FTIR spectra of aqueous SF solution before and after the addition of ethanol and the emulsion system before and after polymerization. Before polymerization, all samples exhibit a sharp peak at 1640 cm^–1^ corresponding to random coil conformation.^34^ This is expected because SF is soluble in the aqueous phase of the emulsion. After polymerization, the peak at 1640 cm^–1^ shifts to 1620 cm^–1^ indicating a conformation transition in SF from random coil to β-sheet structures. The fraction of secondary structures of SF was estimated by separating the hidden peaks at 1620, 1640, 1660, and 1698 cm^–1^ corresponding to β-sheet, random coil, α-helix, and β-turn configurations, respectively,^34−36^ followed by analyzing using a Gaussian model for curve fitting (Figure S3). The β-sheet content of SF in an aqueous solution is 14 ± 2%, while after the addition of the chemical trigger ethanol and stirring for 1 h, it increases to 24.2 ± 0.3% indicating a twofold increase in the β-sheet content that provides emulsion stability and earlier gelation of the emulsion system (inset to Figure 5a). After the addition of C18A into the aqueous phase and mixing at 1400 rpm for 5 min to form a stable emulsion, the β-sheet content of the emulsion system slightly increases to 25% which is attributed to the physical influence of high-speed mixing.^37^ Finally, the β-sheet content after polymerization further increases to 36.2 ± 0.2% which leads to fibroin gelation and, hence, the formation of an SF hydrogel containing dispersed PC18A microgels.

Figure 5b shows XRD profiles of a dried OHG specimen and its components, namely poly (C18A) (PC18A) obtained by UV polymerization, and SF hydrogel. The SF hydrogel component of OHG exhibits an intense peak at 20.6° and two weak peaks at 8.6° and 24.5° corresponding to the β-sheet crystalline spacings of 4.3, 10, and 3.7 Å, respectively.^36^ Moreover, the dispersed PC18A component of OHG shows a crystalline peak at 21.3° corresponding to a Bragg *d*-spacing of 4.2 Å which is typical for the paraffin-like hexagonal lattices formed by the packing of octadecyl (C18) side chains (Scheme 1).^30^ The same peak also appears in the spectrum of OHG revealing that the C18A droplets in the emulsion system are polymerized and formed crystalline domains. Besides this peak, second-order diffraction peaks appear at 25.3°, 23.5°, and 19.6° which we attribute to the β-sheet crystalline structure.^30,38−40^

**Figure 6:**
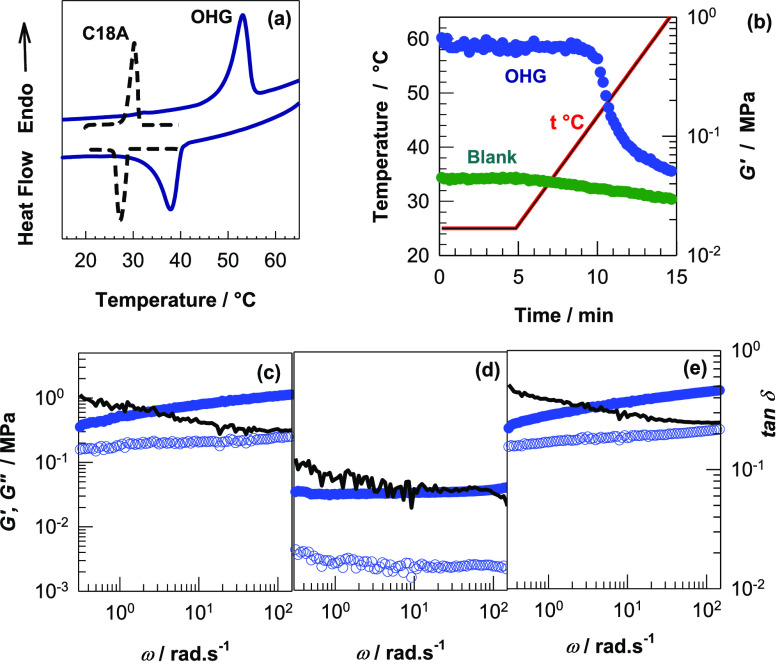
(a) DSC scans of OHG (solid curve) and C18A monomer (dashed curve) during heating from 0 to 70 °C and, back to 0 °C at a heating/cooling rate of 5 °C·min^–1^. Note that no peak was observed at temperatures between 70 and 90 °C. (b) Storage modulus *G*′ (symbols) of OHG and blank samples isothermal at 25° for 5 min, followed by heating from 25 to 65 °C at a rate of 4 °C·min^–1^. The red line represents the temperature–time profile. ω = 6.28 rad·s^–1^. γ_o_ = 0.01. (c–e) Frequency dependence of *G*′ (filled symbols), *G*″ (open symbols), and tan δ (lines) of an OHG specimen at 25 (c), 65 (d), and after cooling back to 25 °C (e). γ_o_ = 0.01.

The solid and dashed curves in Figure 6a present DSC scans of a swollen OHG sample and C18A monomer, respectively, during a heating–cooling cycle between 0 and 70 °C. OHG exhibits melting (*T*_m_) and crystallization temperatures (*T*_cry_) at 53 and 38 °C, respectively, which are typical for semicrystalline PC18A.^30^ Moreover, the melting peak of the C18A monomer at 30 °C does not appear in the DSC scan of OHG supporting its complete conversion to PC18A microgels. Calculations from the area under the melting peak of OHG specimens reveal that the degree of crystallinity *f_cry_*, i.e., the fraction of C18A units forming crystalline domains is 26 ± 1% as compared to 32 ± 4% for PC18A obtained by UV polymerization of C18A. Thus, the confinement of PC18A chains in micron-sized particles does not significantly reduce their alignment to form crystalline domains.

Rheological measurements were conducted to demonstrate the temperature sensitivity of viscoelastic properties of OHGs. Figure 6b shows the storage modulus *G*′ (symbols) of OHG and blank samples at 25° for 5 min, followed by heating to 65 °C at a rate of 4 °C·min^–1^. The modulus of OHG remains constant at 0.6 MPa up to 44 °C while it starts to decrease at higher temperatures and approaches a plateau value of 0.05 MPa at 65 °C, which is close to *G*′ of the blank specimen (0.03 MPa). Thus, a 10-fold decrease in the modulus occurs upon heating from below to above the melting temperature *T*_m_. In contrast, the blank specimen exhibits a temperature-independent profile reflecting the role of the dispersed PC18A phase in the temperature sensitivity of OHG. Figure 6c–e shows frequency dependences of *G*′ (filled symbols), the storage modulus *G*″ (open symbols), and loss factor tan δ (= *G*″/*G*′, lines) of an OHG specimen at 25 (c), 65 (d), and after cooling back to 25 °C (e). At 25 °C, *G*′ increases while tan δ decreases with increasing frequency, indicating the increasing elastic character of OHG at short experimental time scales. This is attributed to the existence of hydrophobic associations in the microinclusions of OHG. Because the degree of crystallinity *f*_cry_ of the OHG is 26 ± 1%, the noncrystalline portion of C18A is around 74% that form hydrophobic associations between the PC18A chains acting as weak cross-links and contributing to the storage modulus at high frequencies. At 65 °C, *G*′ of OHG significantly decreases due to the melting of C18 crystals in the dispersed phase resulting in a lower physical cross-link density. Interestingly, *G*′ of OHG at 65 °C, i.e., above *T*_m_ is almost frequency-independent, and tan δ is less than that measured below *T*_m_ over all frequencies. This indicates that although OHG in the molten state exhibits a low storage modulus, i.e., a low cross-link density, it shows a more elastic character than in the crystalline state. Previous studies on SF hydrogels formed via β-sheet crystals show that their dynamic moduli are independent of the frequency,^36,41^ as the present OHG above *T*_m_. Thus, the melting of C18 crystals and dissociation of hydrophobic associations above *T*_m_ results in SF hydrogel dominating the viscoelasticity of the OHG. Figure 6c–e also shows the reversibility of the viscoelastic properties of OHG during the heating–cooling cycle. For instance, *G*′ of OHG at ω = 10 rad·s^–1^ decreases from 0.3 to 0.05 MPa upon heating from 25 to 65 °C, while cooling back to 25 °C recovers the initial modulus. This reversibly switchable viscoelasticity of OHG due to the semicrystalline microgel inclusions in response to a temperature change is a prerequisite for the thermally induced shape-memory effect.

Mechanical properties of OHG and blank samples in their equilibrium swollen state were investigated by uniaxial compression tests (Figure S4). OHG exhibits 15-fold higher Young’s modulus *E* (1.5 ± 0.5 vs 0.10 ± 0.02 MPa) and 2-fold higher fracture stress σ_f_ (0.40 ± 0.06 vs 0.12 ± 0.02 MPa) as compared to the blank sample highlighting the effect of semicrystalline microinclusions. However, the high degree of crystallinity created in OHG makes it a brittle material with no stretchability and a low compressibility of around 20%. In the following section, we show that the mechanical performance of OHG can be significantly improved by including noncrystallizable monomer units in the dispersed PC8A phase, and a flexible polymer network in the continuous SF hydrogel phase.

### 2.3 Improving Mechanical Properties of OHG

A sol-to-gel transition of aqueous SF solutions occurs by self-assembly of globular SF molecules via intermolecular β-sheet crystals.^42−44^ Owing to the weakness of these cross-links, SF hydrogels rupture at a low strain as also observed in the present study. Oral et al. recently demonstrated that incorporating flexible polymer chains such as poly(*N*,*N*-dimethylacrylamide) (PDMAA) into the SF hydrogel strengthens the intermolecular interactions between SF globules leading to a significant increase in toughness.^38^ To create an efficient energy dissipation mechanism in the continuous SF hydrogel phase of OHG, we included *N*,*N*-dimethylacrylamide (DMAA) monomer together with *N*,*N*′-methylenebisacrylamide (BAAm) cross-linker in the aqueous phase to create an interconnected SF/PDMAA network.^38^ Moreover, although supramolecular semicrystalline hydrogels such as those based on PC18A also exhibit a brittle nature, incorporation of a weak hydrophobe significantly improves their mechanical performances.^31^ To improve the mechanical properties of OHG, noncrystallizable lauryl methacrylate (C12M) units was included in the dispersed PC18A phase to decrease its crystallinity and increase the chain mobility of chains.

Thus, C12M and DMAA monomers and BAAm cross-linker were included in the emulsion system to generate modified OHGs with improved mechanical properties. The addition of C12M into the optimized emulsion system discussed above also produced a stable emulsion with droplet diameters increasing from 18 ± 6 to 31 ± 11 μm with increasing C12M content from 0.3 to 32 mol % (Figure S5). Moreover, incorporation of DMAA up to 7.5 w/v % together with BAAm (1 mol % of DMAA) in the aqueous phase did not change the droplet diameter of the emulsion, and it remained at around 13 μm (Figure S6). Thus, the emulsion system with the optimized conditions could also be used for the preparation of OHGs consisting of a continuous SF/PDMAA hydrogel phase containing C18A/C12M copolymer microinclusions (Figures S7 and S8). Three sets of gelation experiments were carried out (Tables S5–S7). In the first set, DMAA (2.5–7.5 w/v %) was included in the aqueous phase, while in the second set, C12M (0.3–32 mol %) was added into the oil (C18A) phase. In the final set, both 7.5 w/v % DMAA and 32 mol % C12M were included in the aqueous and oil phases, respectively. Hereafter, the concentrations are abbreviated as %.

**Figure 7:**
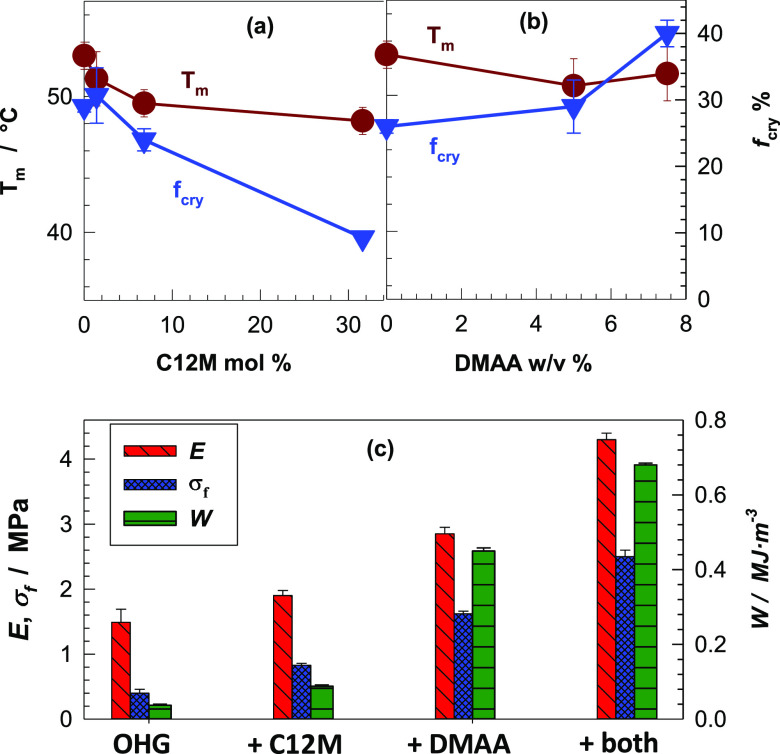
(a, b) *T*_m_ and the degree of crystallinity *f*_cry_ of OHGs depending on C12M (a) and DMAA contents (b). (c) Modulus *E*, fracture stress σ_f_, and toughness *W* of unmodified OHG, and OHGs modified with C12M, DMAA, and both C12M and DMAA. All the mechanical data are the average of at least eight independent measurements. They are displayed in the form of the average value ± standard deviation.

All emulsion systems after UV polymerization resulted in OHGs with a complete gel fraction (Figure S9). The equilibrium water content (EWC) of C12M-modified OHGs remained unchanged at 48 ± 5% with changing C12M content, while DMAA modification resulted in an increase in EWC from 47 to 60% with an increasing amount of DMAA due to its hydrophilicity (Figure S9). Moreover, *T*_m_ of OHGs slightly reduced upon the addition of DMAA or C12M, while the degree of crystallinity *f*_cry_ significantly decreased from 26 ± 1 to 2.3 ± 0.3% after incorporation of 32% C12M (Figure 7a,b), which is attributed to the relatively short alkyl side chains of C12M.^31^

**Figure 8:**
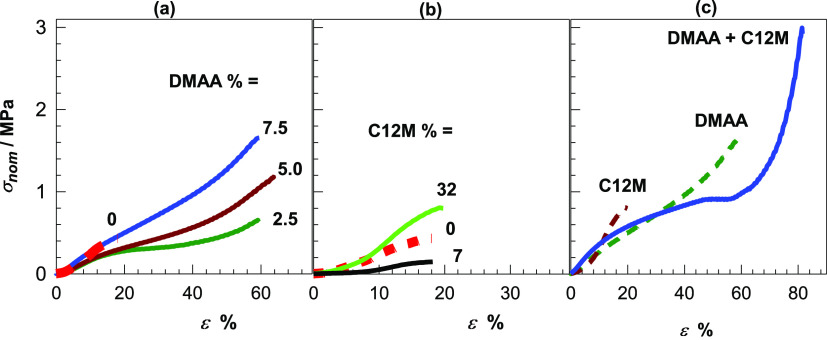
Compressive stress–strain curves of OHGs. (a, b) Effect of DMAA in the aqueous phase (a) and C12M in the oil phase (b). Their amounts are indicated. The dashed red curves represent the data of unmodified OHG. (c) Effect of simultaneous addition 7.5% DMAA and 32% C12M. For comparison, OHGs prepared with only DMAA or C12M addition are also shown.

Figure 8a,b shows compressive stress–strain curves of OHGs with various DMAA and C12M contents, respectively. The bold dashed curves represent the data of unmodified OHG. Introduction of DMAA in the continuous aqueous phase leads to the improvement in all mechanical properties of unmodified OHG, e.g., the compression at break σ_f_ and the toughness *W* increase by 4- and 12-fold, respectively, upon the addition of 7.5% DMAA in the aqueous phase (Figure 7c). Moreover, in contrast to the nonstretchability of unmodified OHG, the OHG prepared with 7.5% DMAA can be stretched to 80% without damage (Figure S10). Although the degree of crystallinity *f*_cry_ significantly decreases upon the incorporation of C12M units into PC18A, no improvement in the mechanical properties of OHG was observed (Figures 7c and 8b). C12M-modified OHGs still behave brittle in both compression and tension as the unmodified OHG. This could be attributed to the increasing flexibility of the chains in the dispersed phase hindering the alignment of C18 side chains to form crystalline domains.

**Figure 9:**
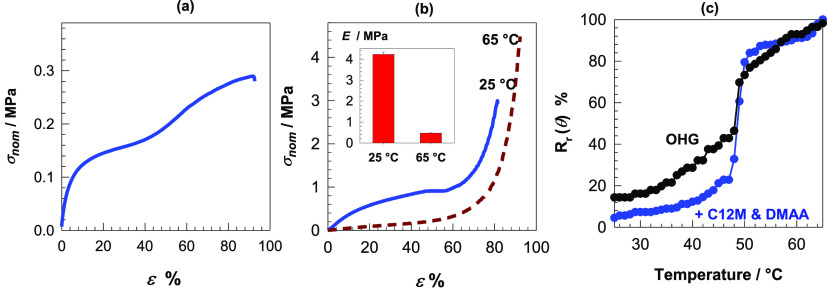
(a, b) Tensile (a) and compressive stress–strain curves (b) of OHG containing both DMAA and C12M. (c) Angular shape-recovery ratio *R*_r_(θ) of OHGs without and with C12M + DMAA shown as a function of temperature.

Interestingly, incorporation of both DMAA and C12M into the aqueous and oil phases, respectively, significantly improved the mechanical properties of unmodified OHG. For instance, the solid curve in Figure 8c shows the compressive stress–strain curve of OHG containing both 7.5% DMAA and 32% C12M, while the dashed curves are the data of OHGs containing the individual C12M and DMAA components. It is seen that both the fracture stress and strain are much higher than those of the OHG components, and simultaneously, Young’s modulus assumes a value of 4.3 ± 0.1 MPa which is maximum over all OHGs (Figures 7c and S11). Moreover, in both compression and tensile curves (Figures 8c and 9a), yielding appears at around 20% strain indicating the occurrence of microscopic damage in the specimens. Figure 8c also shows that the yield strain corresponds to the fracture strain of the brittle C12M-modified OHG. Thus, we may speculate that the C12M-containing PC18A component of OHG in the dispersed phase acts as brittle component fractures at around 20% strain, while the continuous ductile phase consisting of the SF/DMAA network keeps the OHG intact by preventing the crack propagation. In this way, OHG containing both C12M and DMAA sustains 85 ± 3% compression and 95 ± 6% tension without macroscopic damage.

### 2.4 Shape-Memory Function

**Figure 10:**
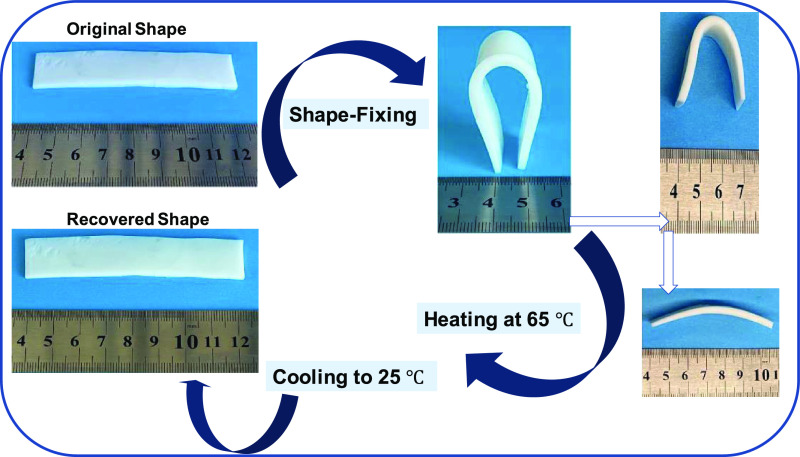
Real-time images demonstrating shape-memory behavior of the OHG with DMAA and C12M. Sample size: 85 × 20 × 2 mm.

All OHGs displayed effective thermally induced shape-memory properties due to the reversibly switchable viscoelasticity (Figure 6c–e) and mechanics in response to a temperature change between below and above *T*_m_. For instance, Figure 9b shows the compressive stress–strain curve of OHG containing both C12M and DMAA units at 25 and 65 °C, i.e., at below and above its *T*_m_. The modulus *E* of OHG around 10-fold decreases (from 4.3 ± 0.1 to 0.47 ± 0.02 MPa) upon heating from 25 to 65 °C. Thus, the OHG specimen softens at 65 °C due to the melting of C18 crystals so that it can easily be deformed into a temporary shape, while cooling below *T*_m_ fixes this temporary shape due to the reformation of C18 crystals. This is illustrated in Figure 10 presenting images of an OHG specimen during shape-fixing and shape-recovery steps. The temporary shape of the specimen remains preserved until heating above *T*_m_ at which the crystalline domains again disappear and hence the polymer chains in OHG return to their most probable initial configuration. Thus, the semicrystalline microgel inclusions in OHG act as the switching segments to fix the temporary shape while the continuous hydrogel phase as the netpoints determine the permanent shape.

To quantify the shape-memory behavior of OHGs, bending tests were conducted on rectangular specimens. In Figure 9c, the angular shape-recovery efficiency *R*_r_(θ) of OHGs without and with C12M + DMAA is shown as a function of temperature. Although both OHGs exhibit a complete shape-recovery ratio at 65 °C, OHG containing both C12M and DMAA is able to fix its temporary shape up to around 40 °C, and the shape-recovery occurs over a narrow range of temperature (47–51 °C) as compared to the unmodified OHG. The improved shape-fixing ability can be explained by the incorporation of the chemically cross-linked DMAA network in the continuous phase of OHG contributing to its entropic elasticity.

### 2.5 Cell Culture Studies of OHGs

**Figure 11:**
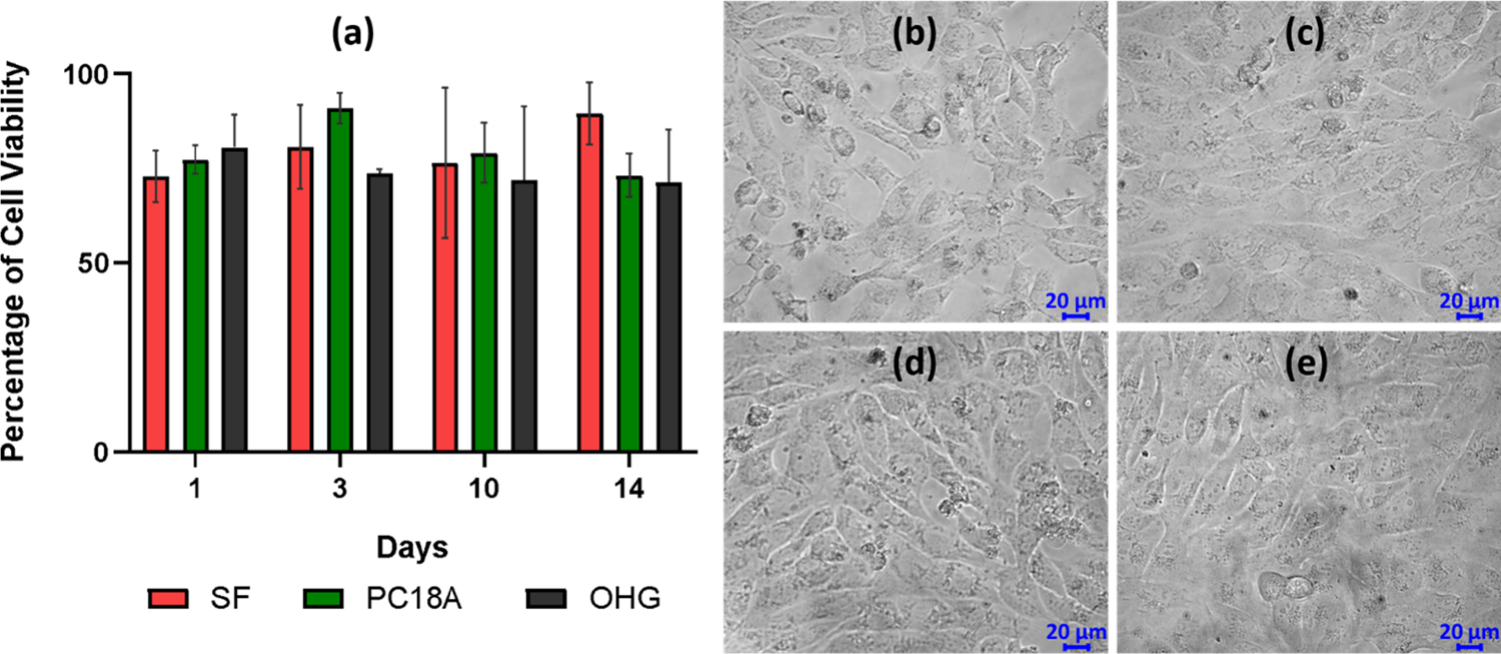
(a) Cytotoxicity analysis of OHG and its SF and PC18A components All data were recorded as the means ±SD for *n* = 3. (b–e) Morphologies of the hFOB cells after exposure to 14-day extracts of SF (b), PC18A (c), OHG (d), and the control (e). Scale bar: 20 μm.

Indirect assay helps to evaluate the cytotoxicity of matrices which may be originated from soluble degradation products, short-chain leachates, etc. The unmodified OHG specimen, and its SF and PC18A components were analyzed on their cytocompatibility. Prior to analysis, extracts from the gel specimens were obtained by placing them in the culture medium for 1, 3, 10, and 14 days of extraction. The cytotoxicity test results after 24 h are shown in Figure 11a for various extraction times. It is seen that there is no significant difference in cell viability (*p* > 0.05) for either material type or increasing incubation time. According to ISO 10993-5, reducing cell viability by more than 30% is considered cytotoxic, so the viability values >75% indicate that none of the materials showed any cytotoxic effect. Previous studies have also demonstrated the nontoxic effect of SF and its combination with C18A.^45^ Optical microscopy images given in Figure 11b–e also reveal that, even after being exposed to 14-day extracts, the morphology of the cells is similar to that of unexposed control cells.

## 3 Conclusions

We designed novel OHGs consisting of an SF hydrogel as the continuous phase and the hydrophobic microinclusions based on semicrystalline PC18A as the dispersed phase. We first prepared a stable oil-in-water emulsion without an external emulsifier by dispersing the C18A monomer in an aqueous SF solution. To stabilize the emulsions for longer times, gelation in the continuous SF phase was induced by the addition of ethanol which is known to trigger the conformational transition in SF from random coil to β-sheet structures. The optimum parameters to obtain a stable emulsion of C18A droplets with a diameter of 12 ± 4 μm were found to be an aqueous 6.5% SF solution containing 17% ethanol in which C18A is dispersed at an o/w volume ratio of 5/5. In the second step, in situ polymerization of C18A droplets in the emulsion system was conducted under UV light in the presence of a photoinitiator to obtain high-strength OHGs with switchable viscoelasticity, shape-memory function, and a good cytocompatibility. The results show that the β-sheet content of SF significantly increases after polymerization leading to the formation of an SF hydrogel containing dispersed semicrystalline PC18A microgels. To further improve the mechanical and shape-memory properties of OHG, DMAA monomer and BAAm cross-linker were included in the aqueous phase to create a continuous hydrogel phase composed of an interconnected SF/PDMAA network. Moreover, noncrystallizable C12M units was also incorporated in the dispersed PC18A phase to decrease its crystallinity and increase the chain mobility of the chains. The modified OHGs exhibit switchable mechanics, a high Young’s modulus (up to 4.3 ± 0.1 MPa), compressive strength (up to 2.5 ± 0.1 MPa), and toughness (up to 0.68 MPa). We should note that the strategy presented here is not limited to C18A monomer which is able to form crystalline domains upon polymerization with *T*_m_ of around 50 °C. *n*-Alkyl (meth)acrylates and *n*-alkyl (meth)acrylamides able to form crystalline domains may also be used in the preparation of OHGs with a tunable phase transition temperature.

## Supporting Information Available

Experimental details including preparation and characterization of the emulsions and OHGs (PDF)
