# Supplementary material for: Silk Fibroin-Based Shape-Memory Organohydrogels with Semicrystalline Microinclusions
Source: ACS Appl Bio Mater. 2023 Mar 16;6(4):1594–603. doi: 10.1021/acsabm.3c00017 (PMC10114111; doi:10.1021/acsabm.3c00017)
Supplement: Supplementary file 1 — mt3c00017_si_001.pdf [file mt3c00017_si_001.pdf]

Supporting Information

Silk Fibroin Based Shape-Memory Organohydrogels with Semicrystalline Microinclusions

*Cigdem Buse ORAL[1], Berkant YETISKIN [1†], Canan CIL [2], Fatma Nese KOK[2], Oguz OKAY[1 *]*

[1] Department of Chemistry, Istanbul Technical University, 34469 Maslak, Istanbul, Turkey

[2] Department of Molecular Biology and Genetics, Istanbul Technical University, 34469 Maslak, Istanbul, Turkey.

[†] Present Address: Department of Chemistry, University of Massachusetts Amherst, Amherst, Massachusetts 01003, United States

* To whom correspondence should be addressed: E-mail: okayo@itu.edu.tr

Table of Contents

Experimental section (S2)
Table S1. Preparation conditions of oil-in-water emulsions at various SF concentrations. (S8)
Table S2. Preparation conditions of oil-in-water emulsions at various ethanol concentrations. (S8)
Table S3. Preparation conditions of oil-in-water emulsions at various o/w ratios. (S8)
Table S4. Preparation conditions of OHG at 6.5 w/v % SF, 17 vol. % ethanol, and o/w = 5/5. (S9)
Table S5. Synthesis condition of OHGs at three different DMAA concentration (S9)
Table S6. Synthesis condition of OHGs at four different C12M concentration in the oil phase. (S9)
Table S7. Synthesis condition of OHG with DMAA and C12M in its aqueous and oil phases, respectively. (S10)
Figure S1. Real-time photographs of a stable emulsion prepared at 6.5% SF concentration. (S10)
Figure S2. Optical images of OHG and blank specimens after extraction in ethanol. (S11)
Figure S3. (a) Typical ATR-FTIR spectra of aqueous SF solution before and after addition of ethanol, and the emulsion system before and after polymerization. (S12)
Figure S4. (a) Compressive stress-strain curves for OHG and blank samples. (b) The angular shape-recovery efficiency $R_r(\theta)$ shown as a function of temperature. (S12)
Figure S5. Optical microscope images of C12M-modified OHGs. (S13)
Figure S6. Optical microscope images of DMAA-modified OHGs. (S13)
Figure S7. Optical microscope image of OHG containing both DMAA and C12M. (S13)
Figure S8. Kernel Density Estimation functions (KDE) of OHGs modified with DMAA, C12M, and both DMAA and C12M. (S14)

Figure S9. Equilibrium water content EWC and the gel fraction W_g plotted against the concentrations of C12M and DMAA. (S14)
Figure S10. Stress strain curves of OHGs prepared at various DMAA contents. (S15)
Figure S11. Images of unmodified and modified OHGs under various strains ε as indicated. (S16)

EXPERIMENTAL SECTION

Materials. *Bombyx Mori* silkworm cocoons were obtained from Kozabirlik (Agriculture Sales Cooperative for Silk Cocoon, Bursa, Turkey). Lithium bromide (LiBr, Merck, 99%), sodium carbonate (Na_2CO_3, Merck, 99.9%), n-octadecyl acrylate (C18A, Sigma-Aldrich, 97%), lauryl methacrylate (C12M, Sigma-Aldrich, 96%), N,N-dimethylacrylamide (DMAA, Sigma-Aldrich, 99%), N,N′-methylenebis(acrylamide) (BAAm, Sigma-Aldrich, 99%), 2-hydroxy-4′-(2-hydroxyethoxy)-2-methylpropiophenone (Irgacure 2959, Sigma-Aldrich, St. Louis, MO), ethanol (Merck, ≥ 99.9%) and polyethylene glycol (PEG-10000, Sigma-Aldrich, 10,000 $g.mol^{-1}$) were used as received. Fluorescein isothiocyanate isomer I (FITC, Sigma-Aldrich, ≥ 90%) and Nile red (Sigma-Aldrich) were used for staining of the hydrogel and the organogel parts of OHGs. Cell culture studies were conducted using human osteoblast cell lines (hFOB, ATCC, CRL11372). Fetal bovine serum (FBS, F7876) was purchased from Sigma-Aldrich. Dulbecco's Modified Medium (DMEM, P04-04510), Trypsin/EDTA (P10-019100), and penicillin-streptomycin (P06-07100) were purchased from PAN™ Biotech. Cell proliferation and cytotoxicity were evaluated using Cell Counting Kit-8 (CCK-8, CK04, Dojindo Molecular Technologies, Inc.).

Isolation of SF from Cocoons. The isolation of silk fibroin (SF) was performed as reported before. Briefly, around 10 g of *Bombyx mori* silkworm cocoons were cut into small pieces and, after cleaning with distilled water, they were boiled in an aqueous solution of Na_2CO_3 (1 L, 0.02 M) for 1 h to remove sericin proteins. After five-time washing of the SF with 1 L of distilled water at 70 °C for 20 min each, it was dried at room temperature for 2 days. 7 g of dried SF was then dissolved in 9.3 M LiBr solution (35

mL) at 60 °C within 2 h. The SF solution was then dialyzed using a 10,000 MWCO dialysis tubing (Snake Skin, Pierce) for 3 days against water that was changed three times a day. After centrifugation, the final SF concentration was ~5 wt%, which was determined by weighing the remaining solid after drying. Aqueous solutions with a higher SF concentration were prepared by concentrating 5 wt% SF solution via dialysis against aqueous solutions of 15 w/v% poly (ethylene glycol) (PEG, Sigma-Aldrich, molecular weight: ~10,000 g mol^{-1}) using 3,500 MWCO dialysis tubing (Snake Skin, Pierce). All SF solutions were stored at 4 °C and used within 2 weeks.

Preparation of Oil-in-Water Emulsions. The emulsions were prepared by dispersing C18A in an aqueous SF solution containing ethanol. A stock solution of SF at a concentration of 9.51 w/v% was used in the experiments. In the first set of experiments, the amount of ethanol and the o/w volume ratio were fixed at 17 vol. % and 4/6, respectively, while the concentration of SF in the aqueous phase was varied. Typically, to prepare an emulsion at 5 w/v % SF, the stock solution of SF (3.15 mL) was diluted with 1.85 mL of distilled water. Ethanol (1 mL) was then dropwise added to the water phase and mixed for 1 h at 40°C and 100 rpm. C18A (3.2 g, or 4 mL) was added to the aqueous phase under 1400 rpm for 15 minutes. The experiments were also carried out in the absence of SF as a control to highlight its emulsifying performance. In the second set, SF concentration and the o/w volume ratio were fixed at 6.5 w/v % and 4/6, respectively, while ethanol content in the aqueous phase was varied. In the final set, both the amounts of SF and ethanol were fixed at 6.5 w/v % and 17 vol. %, respectively, while the o/w volume ratio was varied. The details of the synthesis parameters are given in Tables S1-S3.

The emulsions containing C12M and DMAA monomers were prepared at a fixed o/w volume ratio of 5/5, and at SF and ethanol concentrations of 6.5 w/v % and 17 vol. %, respectively, as described above. C12M mixed with C18A was used as the oil phase of the emulsions at various C12M contents. Moreover, DMAA together with BAAm as a cross-linker (1 mol % of DMAA) were added into the aqueous SF solutions before the preparation of the emulsions.

Preparation of OHGs. OHGs were prepared by adding C18A monomer containing Irgacure photoinitiator (0.2 mole % of C18A) into an aqueous 6.5 w/v % SF solution containing 17 vol. % ethanol at a fixed o/w volume ratio of 5/5, followed by UV polymerization at 23±2 °C for 24 h. Typically, to prepare the aqueous phase of OHG, 3.42 mL of aqueous 9.51 w/v% SF stock solution was diluted with 0.75 mL of distilled water and then, 0.83 mL of ethanol were added slowly to this solution to make the total volume of water phase 5 mL. The aqueous phase was then stirred at 100 rpm for 1 h at 40°C and. Separately, the oil phase was prepared by dissolving 0.2 mol % of Irgacure 2959 photoinitiator in 4 g of C18A monomer. The oil phase was then added into the aqueous phase drop by drop under continuous stirring at 1400 rpm for 5 min at 40°C. The milky white emulsion was transferred into plastic syringes of 1 and 50 mL in volumes and they were placed into the UV reactor at 23±2 °C for 24 h. After polymerization, OHG specimens were placed in an excess of ethanol for 3-4 days by replacing ethanol every day to remove C18A which is a good solvent for C18A. They were then placed in an excess of water for 3-4 days until attaining the swelling equilibrium. The OHGs were dried in a vacuum oven (Nucleus, NVE) at 37 °C for 3-4 days.

Characterization of the Emulsions and OHGs. The emulsions were characterized by the creaming index (CI) as detailed above, and by optical microscopy. After preparation of the emulsions, analysis of randomly selected 100 emulsion droplets were conducted using an optical microscope (Olympus, CX31) at 10x magnification. Real-time photographs of the emulsions were taken by QImaging MicroPublisher 3.3 RTV camera, and the size distribution of the emulsion droplets was determined by measuring the diameter using ImagePro Plus software. Distribution of the emulsion droplets was presented with histogram graphs that were fitted by utilizing kernel density estimation (KDE) which is similar to Gaussian distribution. In KDE, probability density function is formed through non-parametric

calculations while in Gaussian distribution, it is obtained through parametric way. For the unevenly and randomly distributed emulsion droplets, KDE was the best way to fit histogram data.

To determine the conformation of SF, ATR-FTIR measurements were conducted on an Agilent Technologies Cary 630 ATR-FTIR spectrophotometer as detailed before. In short, the Amide-I region of the spectra was deconvolved by Gaussian model after a linear baseline correction by using PeakFit software (Version 4.12, SeaSolve Software Inc.). Then, the band positions at 1620, 1640, 1660 and 1698 cm^{-1} corresponding to β-sheet, random coil, α-helix and β-turn respectively, were fixed for curve fitting while allowing their widths and heights to vary (Fig S3). Differential scanning calorimetry (DSC) measurements were performed on a Perkin Elmer DSC 4000 instrument under nitrogen atmosphere. The specimens of about 10 mg were put in aluminum pans and then they were scanned between 0 - 65 °C. Melting enthalpies ΔH_m were calculated from the DSC curves and the degree of crystallinity f_{cry}, i.e. the fraction of C18A units forming crystalline regions was determined as reported before. XRD measurements were conducted on dried specimens on a PANalytical X-Pert PRO multi-purpose diffractometer using Ni-filtered CuKα (λ = 0.15418 nm) radiation at 45 kV and 40 mA in the range of 2θ = 5–40°. To visualize the hydrogel and the organogel components of OHG, confocal laser scanning microscopy (CLSM, Nikon C2, Japan) was used after their staining with FITC and Nile Red, respectively.

The equilibrium water content *EWC* of OHGs was calculated as $EWC = 1 - 1/q_w$ where q_w is the equilibrium weight swelling ratio of OHGs with respect to dry state. Moreover, the gel fraction W_g was calculated using the equation

$$W_g = \frac{m_{dry}}{m_o\, C_o} \qquad (1)$$

where m_o is the mass of OHG after preparation, and C_o is concentration of SF and the hydrophobic monomer in the emulsion system.

Rheological measurements were conducted on a Bohlin Gemini 150 Rheometer system equipped with a Peltier device that controls the temperature. A parallel plate geometry (diameter = 20 mm) and a water trap were used. Frequency (ω) sweep tests were conducted at a fixed strain amplitude γ_0 of 0.1%. Temperature dependent viscoelastic properties of OHGs were determined by heating the OHG specimens from 25 to 65 °C at a fixed rate of 4 $°C \cdot min^{-1}$ at $\omega = 6.28$ $rad \cdot s^{-1}$ and $\gamma_0 = 0.1\%$.

Mechanical performances of OHGs were performed at 23±2 °C and at a test speed of 5 $mm \cdot min^{-1}$ using a Zwick-Roell universal test machine equipped with a load cell of 500 N. Young's modulus E was determined from the stress-strain curves between 2-4% compression. The stress was represented by its nominal value σ_{nom} (the force per cross-sectional area of undeformed specimen), while strain was given by ε (sample length change relative to its initial length).

Shape-memory tests were performed on the OHGs via bending tests. Rectangular OHG specimens (85×20×2 mm) were heated up to 65 °C and deformed at this temperature to a bended temporary shape with a deformed angle θ_d. They were then exposed to 25 °C for 5 minutes to fix this temporary shape under load. After unloading, fixed angles θ_f of the specimens were measured to determine the shape fixity ratios R_f of OHGs. Finally, OHG specimens were gradually heated from 25 °C to 70 °C and recovery angles θ_r in each 1-3 °C temperature intervals were recorded, and shape recovery ratios R_r were calculated. The shape fixity ratio R_f, and the temperature dependent shape-recovery ratio R_r of OHGs, respectively, were calculated as

$$R_f = \frac{\theta_f - 180}{\theta_d - 180} \quad (2)$$

$$R_r = \frac{\theta_d - \theta_r}{\theta_d - 180} \quad (3)$$

where θ_d, θ_f, and θ_r are the deformed angle under load, fixed angle after unloading, and recovered angle upon heating, respectively.

Cell Culture Studies. Human osteoblast cell lines, hFOB (ATCC, CRL11372) were cultured in Dulbecco's Modified Eagle Medium (DMEM) containing 10 vol. % fetal bovine serum (FBS) and 1 vol. % penicillin-streptomycin and incubated in a humidified atmosphere with 5% CO_2 at 37 °C (Series C, Binder). The cells were collected by trypsinization-centrifugation step (1000 rpm) and suspended in fresh DMEM medium for indirect contact interaction analysis as stated by ISO 10993-1 (2016) and ISO 10993-5 (2009).

For indirect interaction, OHG specimens and its SF and PC18A components were cut into 3 mm thick pieces and sterilized under UV light for 60 min. They were then immersed in the culture medium to extract any leachable components from the specimens, during which subsamples were collected after 1, 3, 10, and 14 days of extraction. Separately, cells suspended in the fresh culture medium were seeded on the 96-well plate (1×10^4 cells/well) and incubated for 24 h. After they reached ca. 80% confluency, the subsamples collected at different time intervals were added into the wells, and left in contact for another 24 h. CCK-8 was used to determine the cell number based on mitochondrial activity for all groups. After 24 h of incubation, the cell culture medium in the wells was removed, and the fresh one, including 10 % CCK-8 solution, was added. After 3 h of incubation at 37°C (CO2 5 %), the optical density values were measured at 450 nm by a microplate reader (Benchmark Plus, Bio-Rad). The cells were also visualized by light microscope (Axiovert A1, Zeiss) to determine any morphological difference.

All data were recorded as the means ±SD for n=3. Statistical analysis was performed on GraphPad prism 8.0.1 by two-way analysis of variance (ANOVA) test. Values of $p<0.05$ were considered statistically significant.

Table S1. Preparation conditions of oil-in-water emulsions at various SF concentrations. Ethanol = 17 vol. %. o/w = 4/6. The stock SF solution contains 9.51 w/v % SF.

**SF w/v% in water phase**		**0**	**5.0**	**5.5**	**6.0**	**6.5**	**7.0**
Water Σ 6 mL	SF stock (mL)	0	3.15	3.47	3.79	4.1	4.42
	Water (mL)	5	1.85	1.53	1.21	0.9	0.58
	Ethanol (mL)	1	1	1	1	1	1
Oil Σ 4 mL	C18A (mL)	4	4	4	4	4	4

Table S2. Preparation conditions of oil-in-water emulsions at various ethanol concentrations. SF = 6.5 w/*v* %. o/w = 4/6. The stock SF solution contains 9.51 w/v % SF.

**Ethanol vol.% in water phase**		**0**	**13**	**15**	**16**	**17**
Water Σ 6 mL	SF stock (mL)	4.10	4.10	4.10	4.10	4.10
	Water (mL)	1.90	1.10	1	0.95	0.90
	Ethanol (mL)	0	0.80	0.90	0.95	1
Oil Σ 4 mL	C18A (mL)	4	4	4	4	4

Table S3. Preparation conditions of oil-in-water emulsions at various o/w ratios. SF = 6.5 w/*v* %. Ethanol = 17 vol. %. The stock SF solution contains 9.51 w/v % SF.

**o/w ratio**		**2/8**	**3/7**	**4/6**	**5/5**
Water	SF stock (mL)	5.47	4.78	4.1	3.42
	Water (mL)	1.19	1.05	0.9	0.74
	Ethanol (mL)	1.34	1.17	1	0.84
	Total volume (mL)	8	7	6	5
Oil	C18A (mL)	2	3	4	5
	Oil total volume	2	3	4	5

Table S4. Preparation conditions of OHG at 6.5 w/v % SF, 17 vol. % ethanol, and o/w = 5/5. The stock SF solution contains 9.51 w/v % SF.

Water Σ 5 mL	SF stock	3.418 mL
	Water (mL)	0.747 mL
	Ethanol	0.835 mL
Oil Σ 5mL	C18A	5 mL
	Irgacure 2959	5.53 mg

Table S5. Synthesis condition of OHGs at three different DMAA concentration in the aqueous phase. The stock SF solution contains 9.51 w/v % SF.

**DMAA w/v %**		**2.5**	**5**	**7.5**
Water Σ 5 mL	SF stock	3.421 mL	3.421 mL	3.421 mL
	Water	0.614 mL	0.484 mL	0.355 mL
	Ethanol	0.835 mL	0.835 mL	0.835 mL
	DMAA	0.130 mL	0.260 mL	0.389 mL
	BAAm	1.94 mg	3.89 mg	5.83 mg
	Irgacure 2959	0.56 mg	1.13 mg	1.69 mg
Oil Σ 5 mL	C18A	5 mL	5 mL	5 mL
	Irgacure 2959	5.53 mg	5.53 mg	5.53 mg

Table S6. Synthesis condition of OHGs at four different C12M concentration in the oil phase. The stock SF solution contains 9.51 w/v % SF. The amount of C12M is presented as mol % in C18A + C12M mixture.

**C12M mol %**		**0.3**	**1.4**	**7**	**32**
Water Σ 5 mL	SF stock	3.421 mL	3.421 mL	3.421 mL	3.421 mL
	Water	0.744 mL	0.744 mL	0.744 mL	0.744 mL
	Ethanol	0.835 mL	0.835 mL	0.835 mL	0.835 mL
Oil Σ 5 mL	C18A	4.99 mL	4.95 mL	4.75 mL	3.75 mL
	C12M	0.01 mL	0.05 mL	0.25 mL	1.25 mL
	Irgacure 2959	5.53 mg	5.55 mg	5.63 mg	6.1 mg

Table S7. Synthesis condition of OHG with DMAA and C12M in its aqueous and oil phases, respectively. The stock solutions of SF and DMAA contain 9.51 and 7.5 w/v % SF and DMAA, respectively.

Water Σ 5 mL	SF stock	3.421 mL
	Water	0.355 mL
	Ethanol	0.835 mL
	DMAA	0.389 mL
	BAAm	5.83 mg
	Irgacure 2959	1.69 mg
Oil Σ 5 mL	C18A	3.75 mL
	C12M	1.25 mL
	Irgacure 2959	6.1 mg

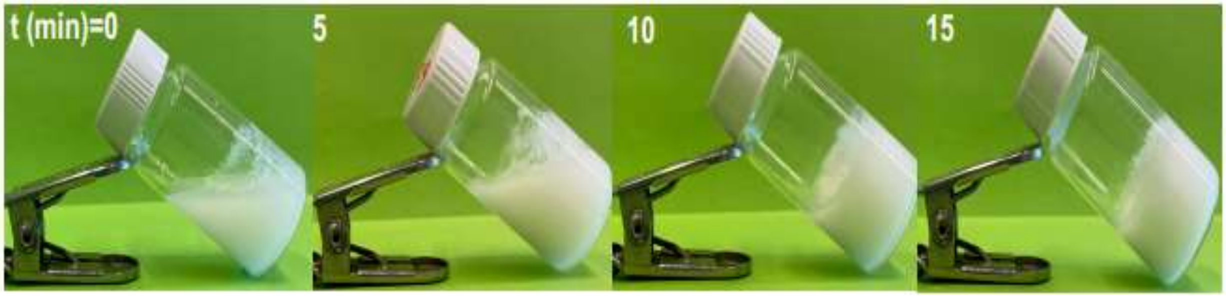

Figure S1. Real-time photographs of a stable emulsion prepared at 6.5% SF concentration. CI = 0. The sol-to-gel transition occurs after 15 min.

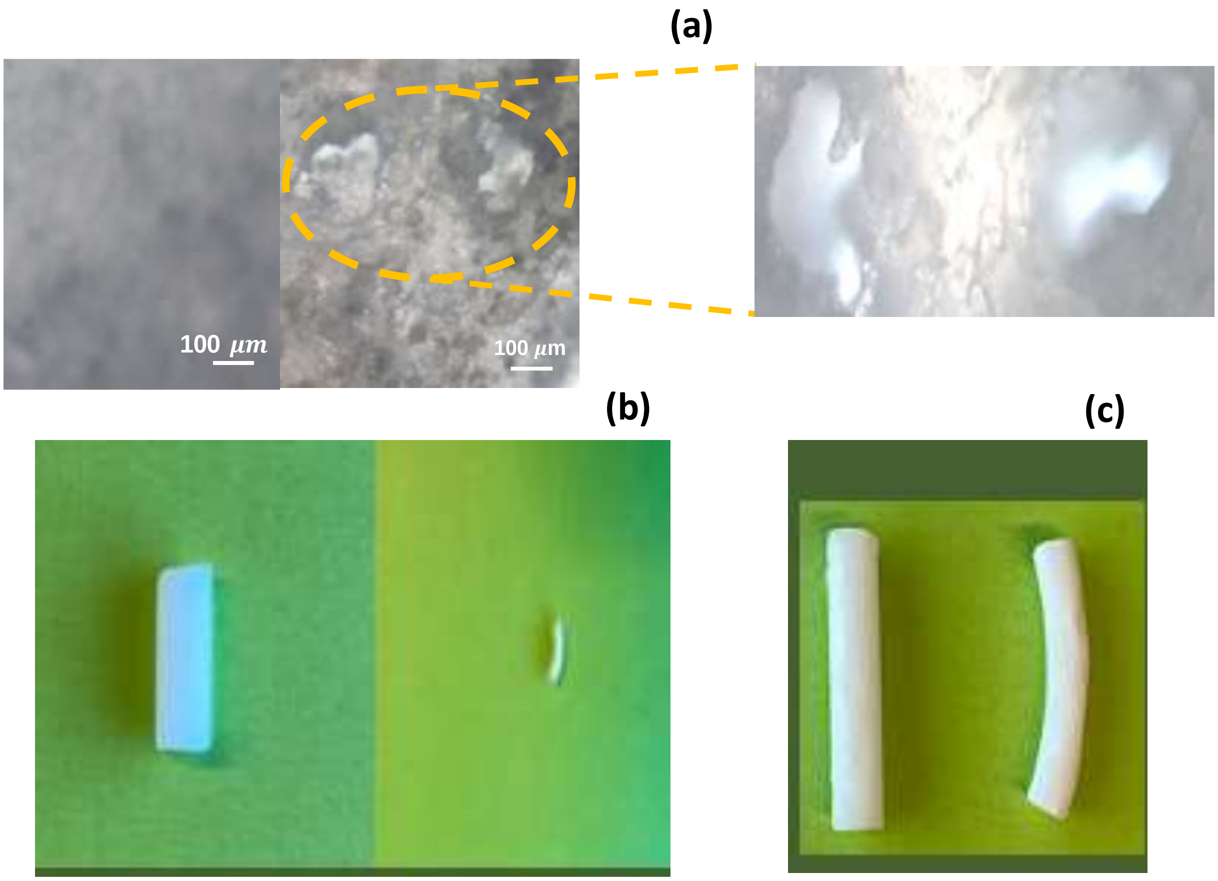

Figure S2. (a) Optical images of OHG (left) and blank specimens (right) after extraction in ethanol. (b, c) Optical images of blank (b) and OHG specimens (c) in water-swollen (each left) and dry states (each right). The blank specimen collapses upon drying due to the shrinkage of the pores.

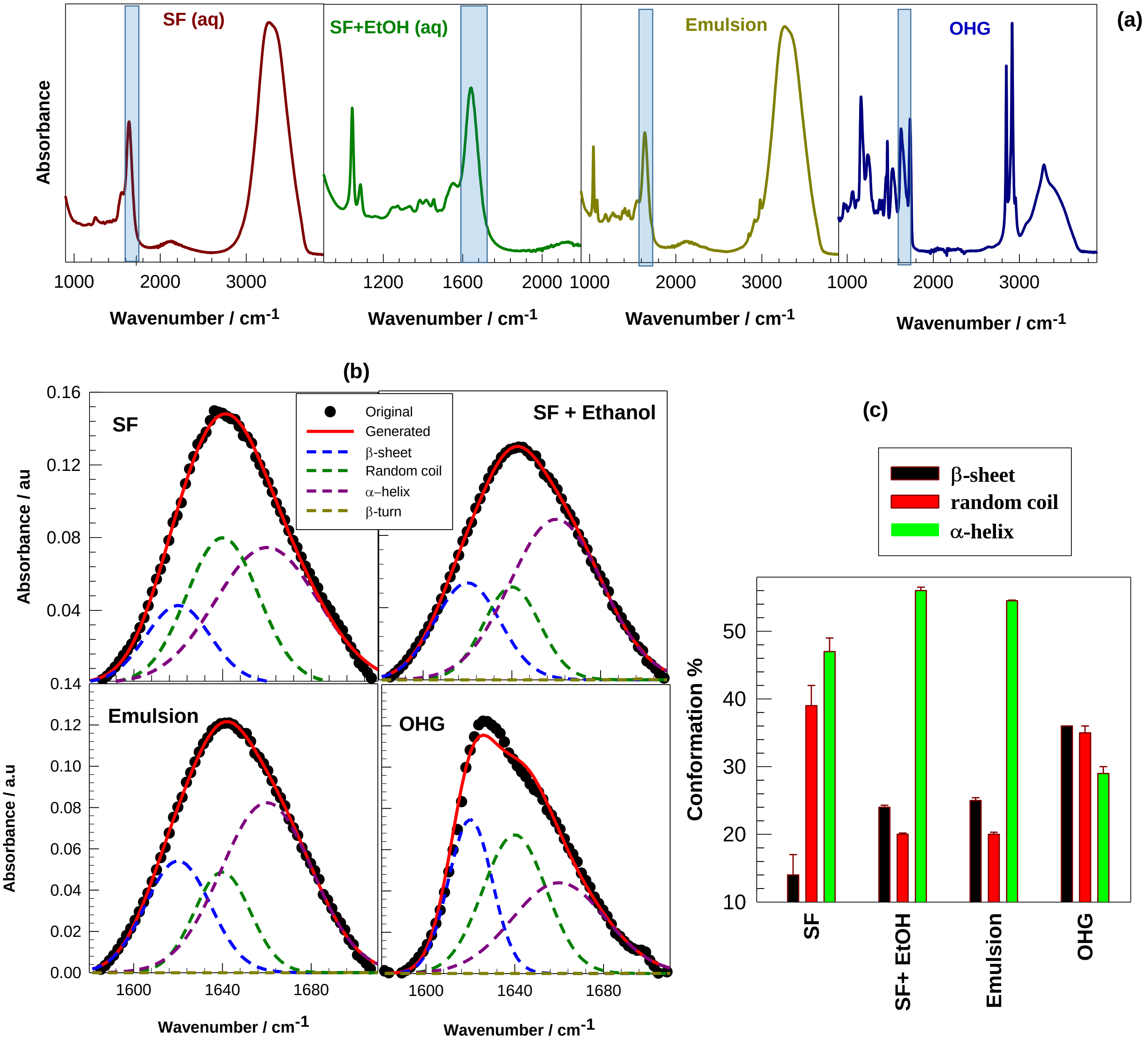

Figure S3. (a) FTIR spectra of aqueous SF solution before and after addition of ethanol, and the emulsion system before and after polymerization, denoted by SF, SF + Ethanol, emulsion, and OHG, respectively. Amide I region is indicated by the blue color. (b) Amide I region of the FTIR spectra. The original data are shown by the filled circles while the results of curve fitting for the original spectrum and hidden peaks are shown by the solid and dashed curves, respectively. (c) The secondary conformation percentages of SF, SF + Ethanol, emulsion, and OHG.

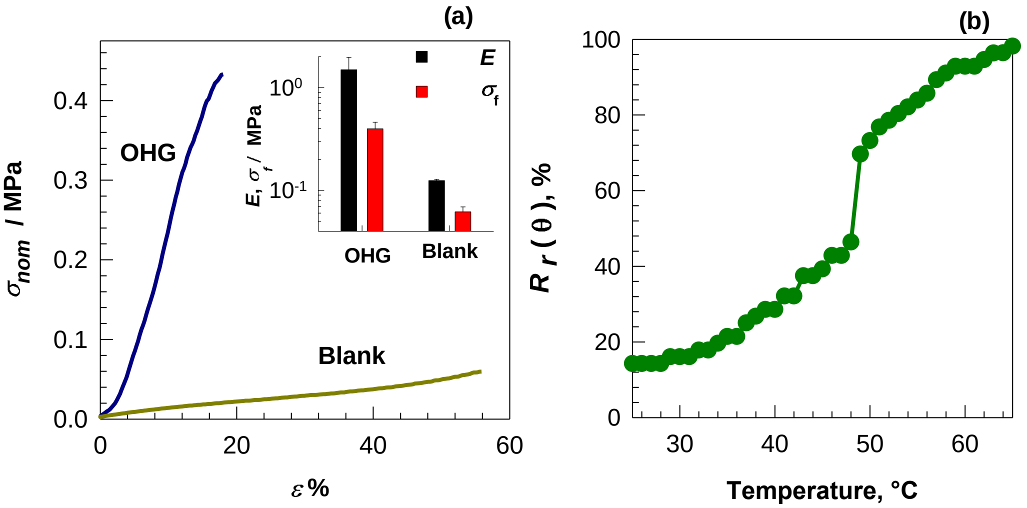

Figure S4. (a) Compressive stress-strain curves for OHG and blank samples. The inset show their Young's modulus E and fracture stress σ_f. (b) The angular shape-recovery efficiency $R_r(\theta)$ shown as a function of temperature.

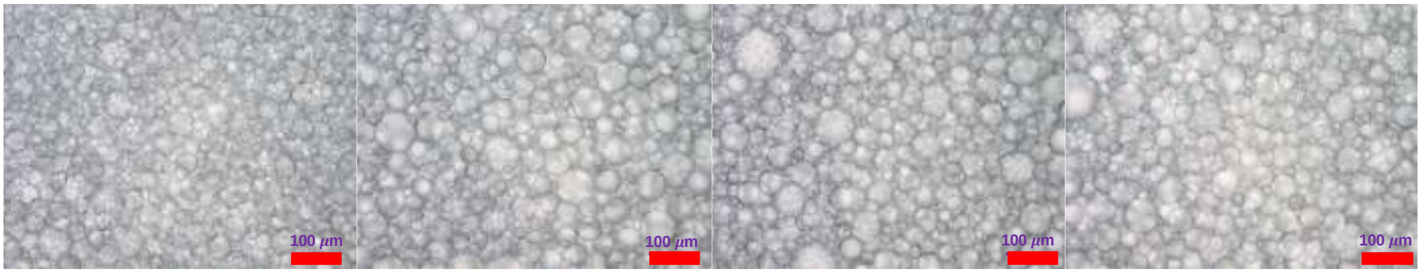

Figure S5. Optical microscope images of C12M-modified OHGs. C12M (from left to right): 0.3, 1.4, 7, and 32 mol %. Scale bar = 100 μm.

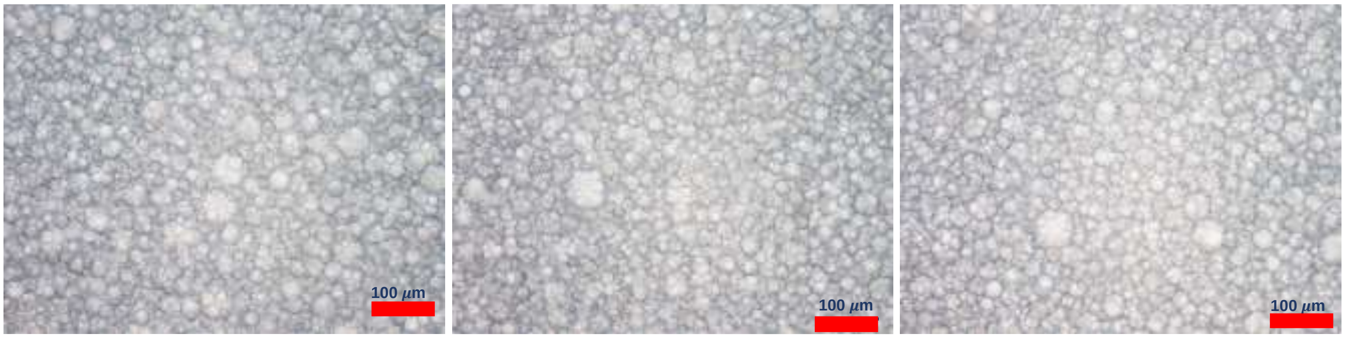

Figure S6. Optical microscope images of DMAA-modified OHGs. DMAA(from left to right): 2.5, 5, and 7.5 w/v %. Scale bar = 100 μm.

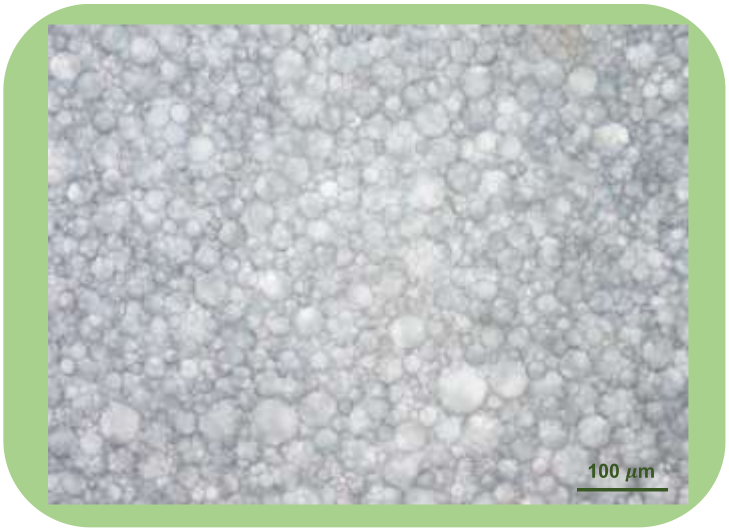

Figure S7. Optical microscope image of OHG containing both 7.5 w/v % DMAA and 32 mol % C12M. Scale bar = 100 μm.

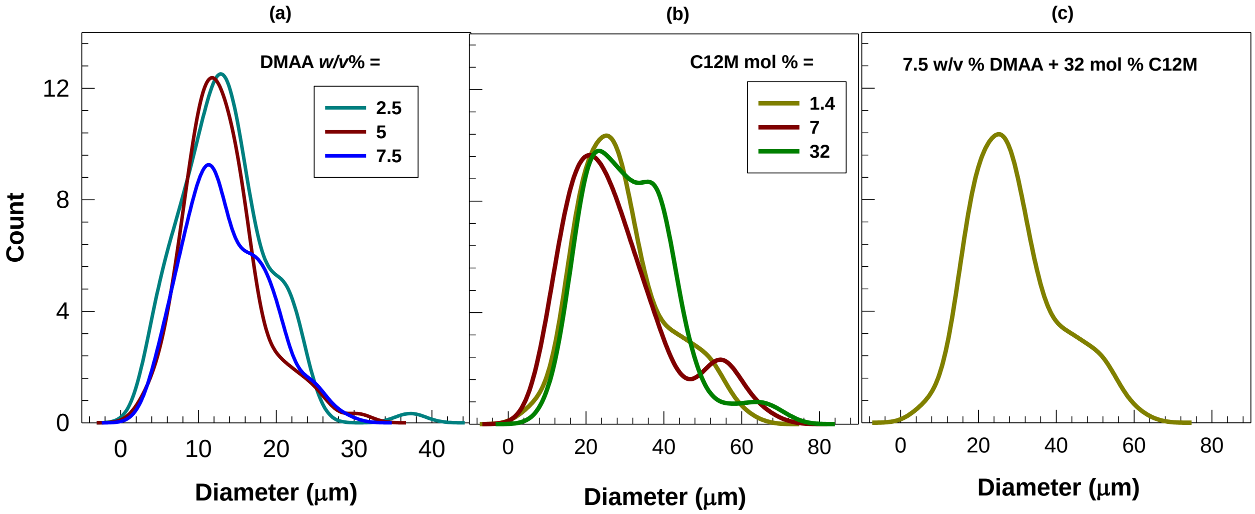

Figure S8. Kernel Density Estimation functions (KDE) of OHGs modified with DMAA (a), C12M (b), and both DMAA and C12M (c).

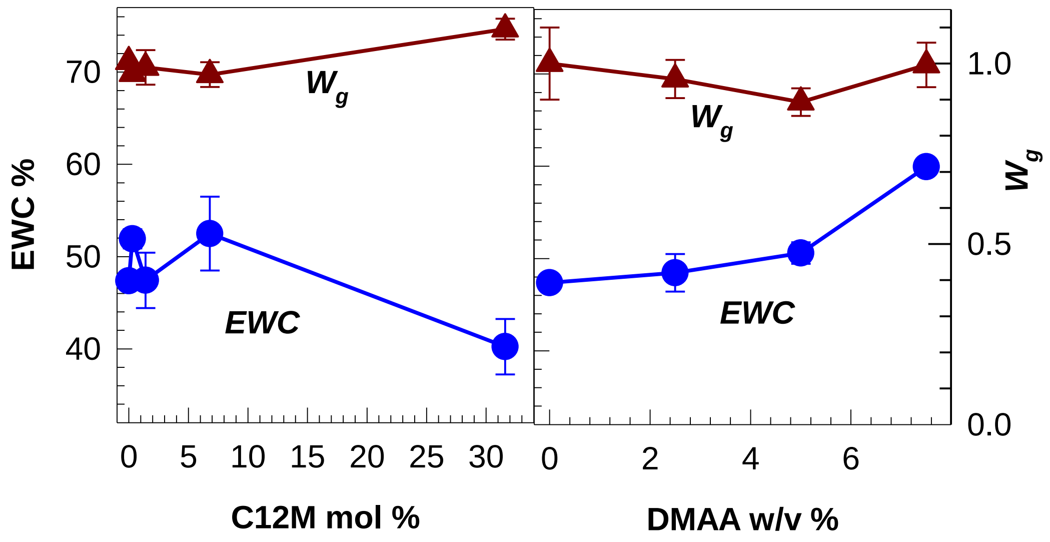

Figure S9. Equilibrium water content EWC (circles) and the gel fraction W_g (triangles) plotted against the concentrations of C12M and DMAA.

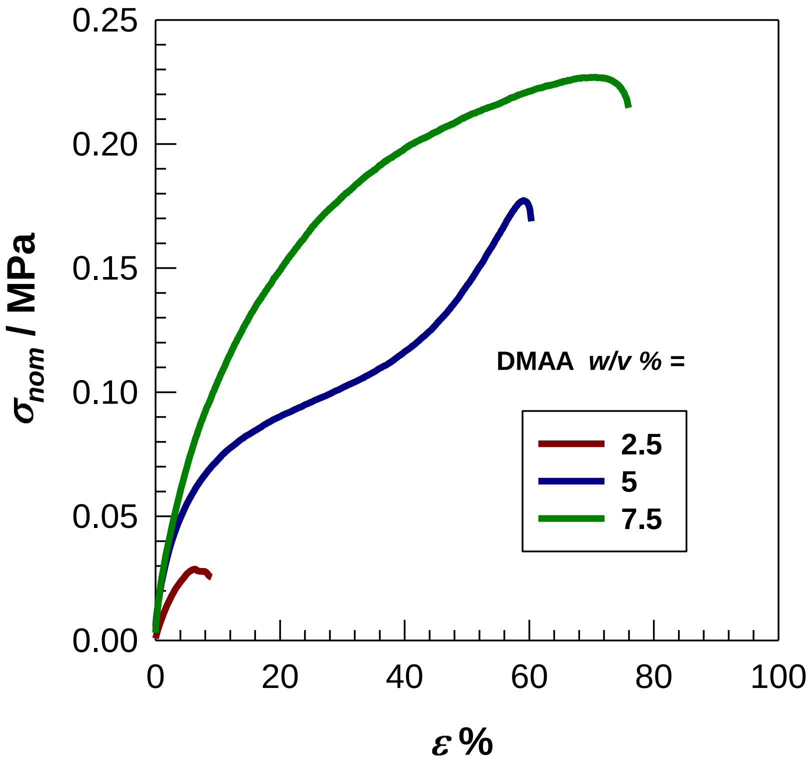

Figure S10. Stress strain curves of OHGs prepared at various DMAA contents as indicated.

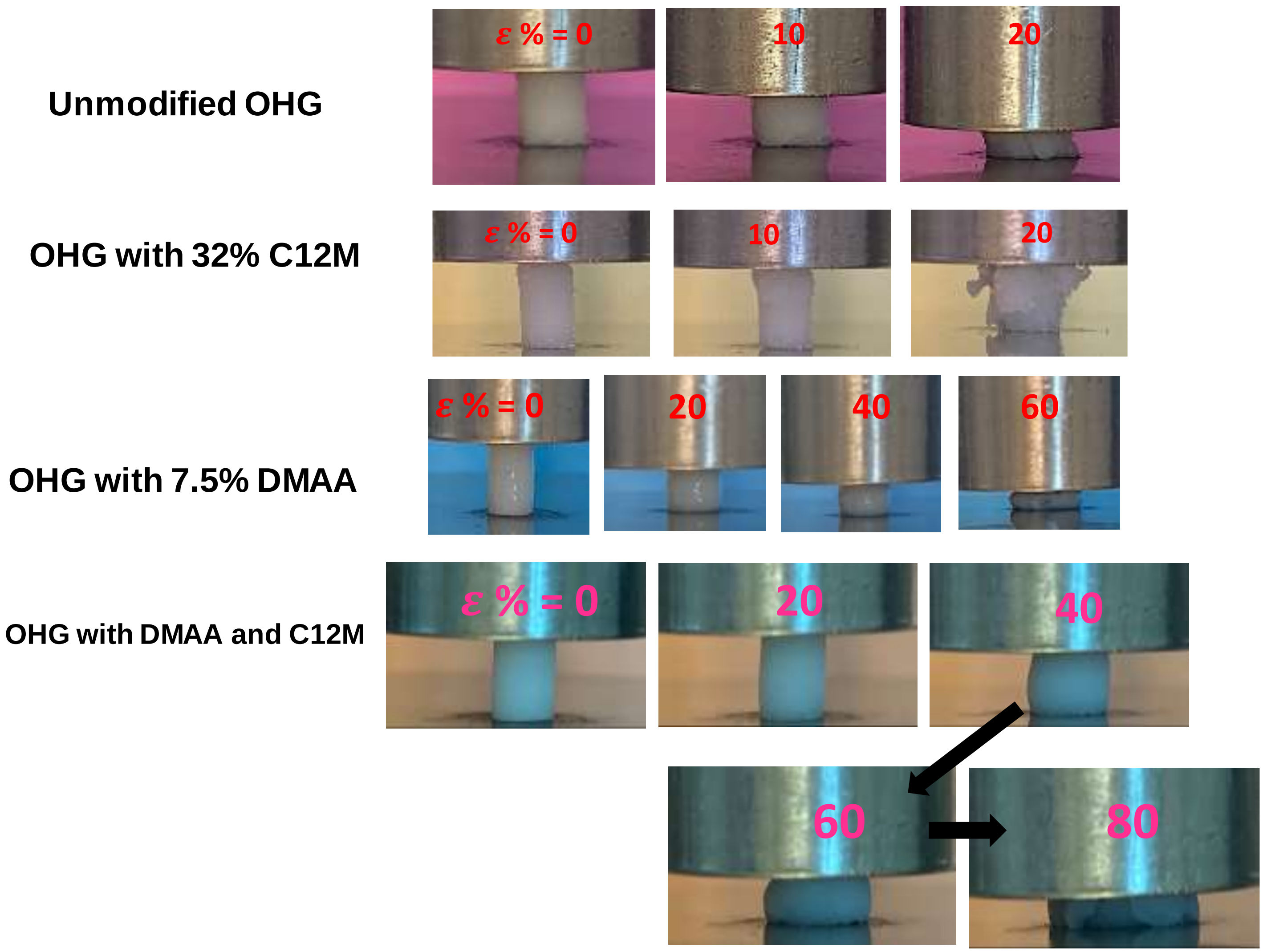

Figure S11. Images of unmodified and modified OHGs under various strains ε as indicated.
